# Supplementary material for: Photorelaxation Pathways of 4-(N,N-Dimethylamino)-4′-nitrostilbene Upon S1 Excitation Revealed by Conical Intersection and Intersystem Crossing Networks
Source: Molecules. 2020 May 9;25(9):2230. doi: 10.3390/molecules25092230 (PMC7248955; doi:10.3390/molecules25092230)
Supplement: Supplementary file 1 [file molecules-25-02230-s001.pdf]

Supporting Information

Photorelaxation pathways of 4-(N,N-dimethylamino)-4'-nitrostilbene upon S_1 excitation revealed by conical intersection and intersystem crossing networks

Ziyue He[a], Ruidi Xue[a], Yibo Lei[a*], Le Yu[a*] and Chaoyuan Zhu[bc*]

[a] *Key Laboratory of Synthetic and Natural Functional Molecule of the Ministry of Education, College of Chemistry & Materials Science, Shaanxi key Laboratory of Physico-Inorganic Chemistry, Northwest University, Xi'an, Shaanxi, 710127, P. R. China.*

[b] *Institute of Molecular Science, Department of Applied Chemistry, and Center for Interdisciplinary Molecular Science, National Chiao Tung University, Hsinchu 300, Taiwan.*

[c] *Center for Emergent Functional Matter Science, National Chiao Tung University, Hsinchu 30010, Taiwan.*

S1 Active orbitals occupation numbers of selected active spaces for DANS................2

S2 The occupation numbers of active orbitals and relative potential energies (in eV) calculated by SA6-CASSCF(18,12) method for the *trans*-, *cis*-, *twist*-DANS and DHP...6

S3 The occupation numbers of active orbitals and relative potential energies (in eV) calculated by SA6-CASSCF(18,12) method for the conical intersections and intersystem crossings..7

S4 The LIICs for optimized CIs and ISCs that calculated by SA6-CASSCF(18,12) method..8

S5 Geometries for SA6-CASSCF(18,12) optimized minima and TS of DANS..........10

S6 Cartesian coordinates for all optimized geometries...10

S1. Active orbitals and occupation numbers of selected active spaces for DANS.

Figure S1a. The SA6-CASSCF(18,12) computed active orbitals of *trans*-S_0 with occupation numbers in parentheses.

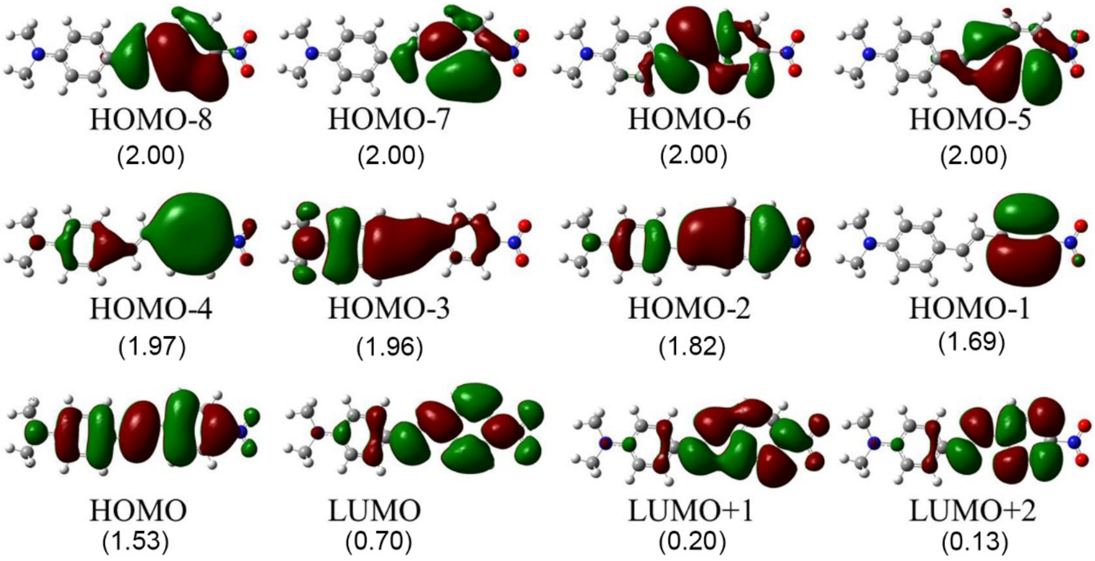

Figure S1b. The SA6-CASSCF(10,8) computed active orbitals of *trans*-S_0 with occupation numbers in parentheses.

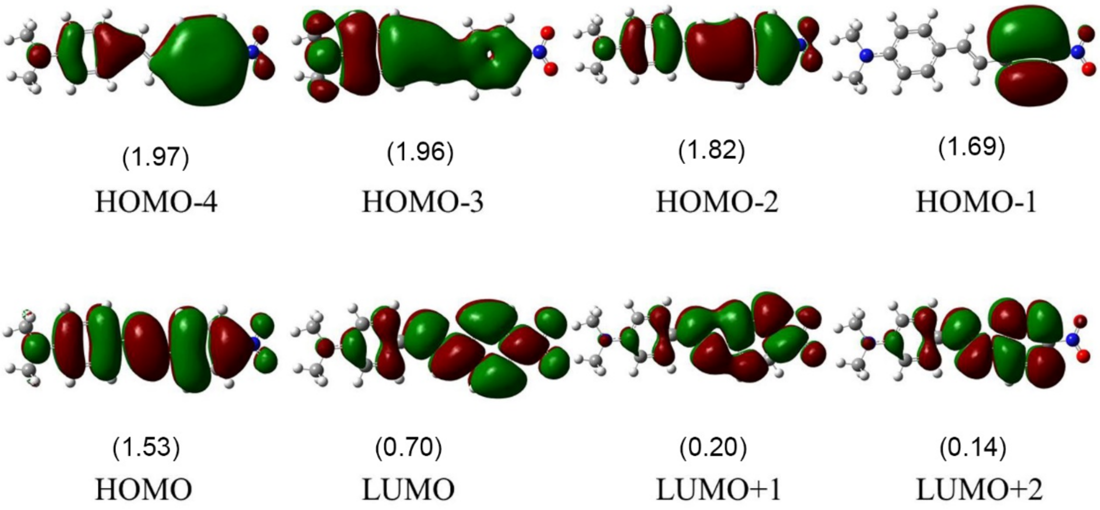

Figure S1c. The SA6-CASSCF(10,10) computed active orbitals of *trans*-S_0 with occupation numbers in parentheses.

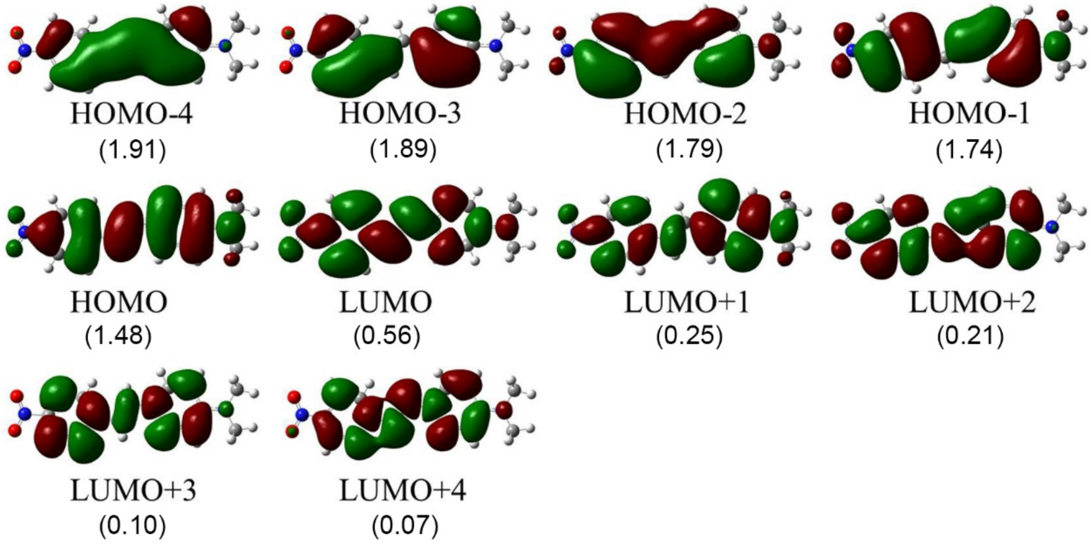

Figure S1d. The SA6-CASSCF(12,12) computed active orbitals of *trans*-S_0 with occupation numbers in parentheses.

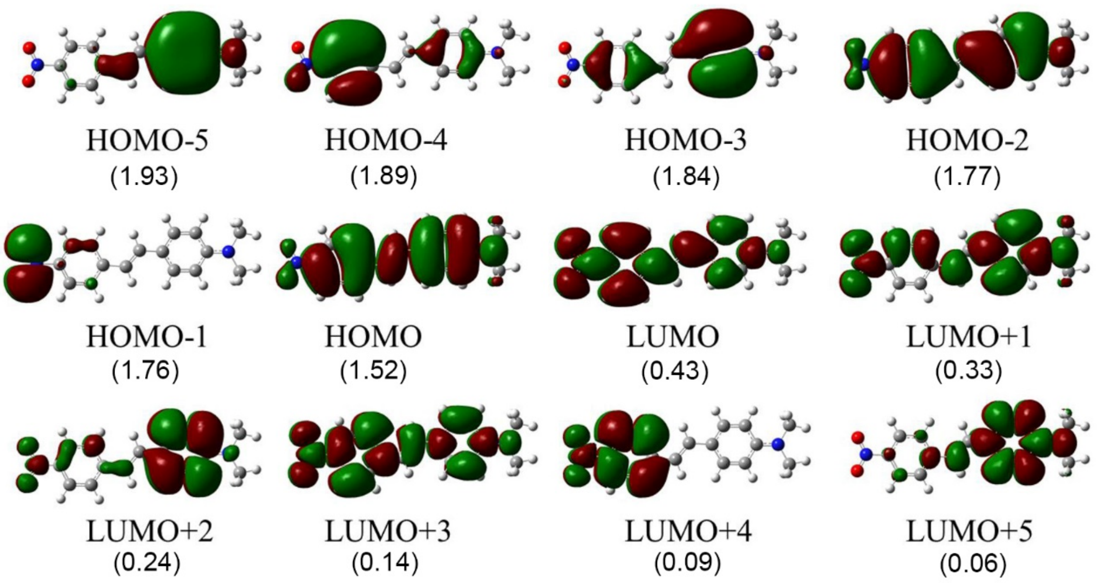

Figure S1e. The SA6-CASSCF(18,12) computed active orbitals of *cis*-S_0 with occupation numbers in parentheses.

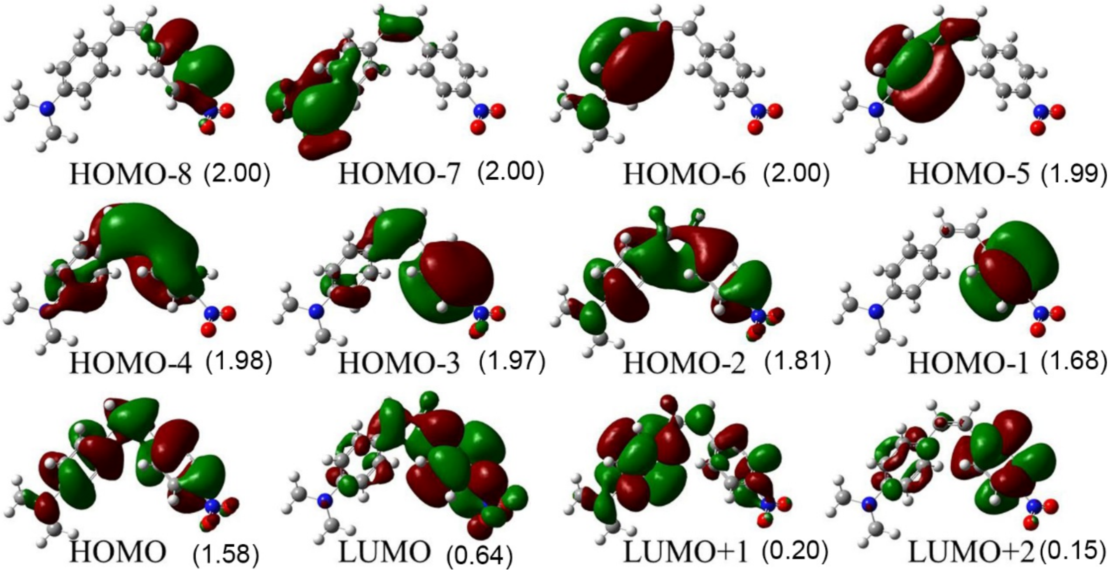

Figure S1f. The SA6-CASSCF(10,8) computed active orbitals of *cis*-S_0 with occupation numbers in parentheses.

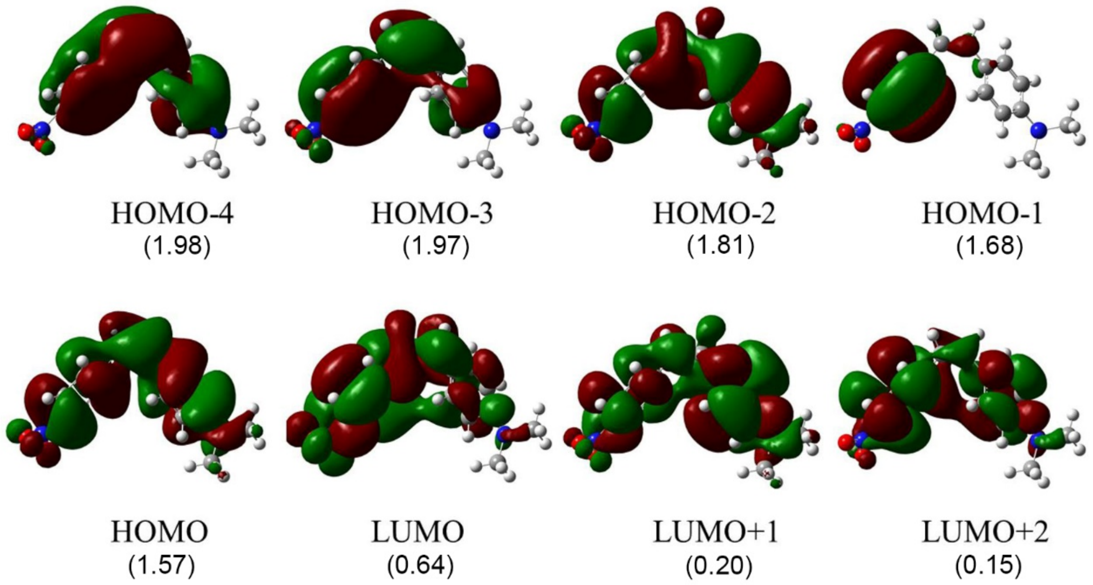

Figure S1g. The SA6-CASSCF(10,10) computed active orbitals of *cis*-S_0 with occupation numbers in parentheses.

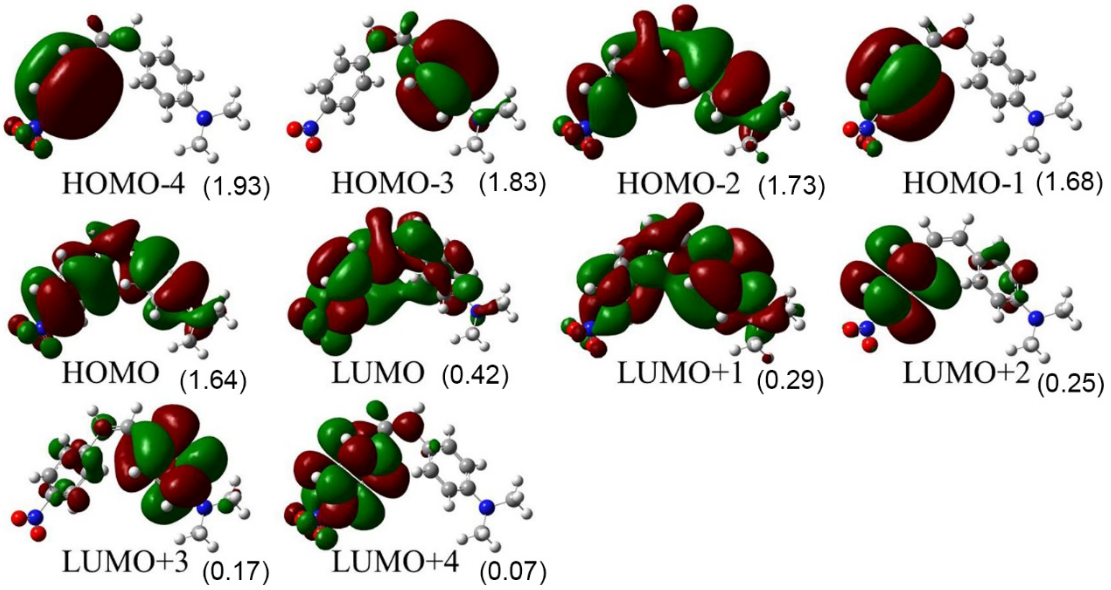

Figure S1h. The SA6-CASSCF(12,12) computed active orbitals of *cis*-S_0 with occupation numbers in parentheses.

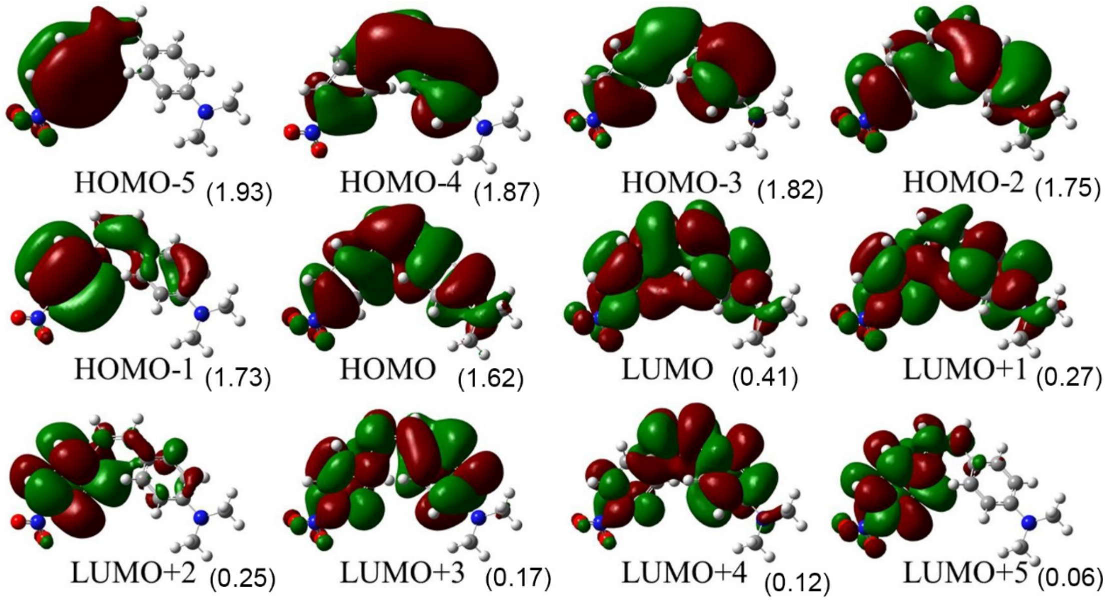

S2. The occupation numbers of active orbitals and relative potential energies for minima.

Table S1. The occupation numbers of active orbitals calculated by SA6-CASSCF(18,12) for the *trans*-, *cis*-, *twist*-DANS and DHP.

	H-8[a]	H-7	H-6	H-5	H-4	H-3	H-2	H-1	H	L	L+1	L+2
trans-S_0	2.00	2.00	2.00	2.00	1.97	1.96	1.82	1.69	1.53	0.70	0.20	0.13
cis-S_0	2.00	2.00	2.00	1.99	1.98	1.97	1.81	1.68	1.58	0.64	0.20	0.15
DHP-S_0	2.00	2.00	1.99	1.99	1.99	1.97	1.76	1.74	1.42	0.67	0.27	0.19
TS-S_0	2.00	2.00	2.00	1.99	1.99	1.99	1.83	1.75	1.20	0.89	0.22	0.13
trans-S_1	2.00	2.00	1.99	1.99	1.96	1.91	1.73	1.63	1.61	1.05	0.09	0.05
twist-S_1	2.00	2.00	2.00	1.99	1.99	1.99	1.83	1.75	1.22	0.88	0.22	0.13
DHP-S_1	2.00	2.00	1.99	1.99	1.98	1.97	1.90	1.73	1.11	0.89	0.34	0.10
trans-T_1	2.00	2.00	1.99	1.99	1.94	1.94	1.71	1.65	1.63	1.03	0.07	0.07
twist-T_1	2.00	2.00	2.00	1.99	1.99	1.99	1.84	1.74	1.19	0.91	0.21	0.15
DHP-T_1	2.00	2.00	1.99	1.98	1.98	1.97	1.90	1.68	1.18	0.95	0.26	0.11
trans-T_2	2.00	2.00	1.99	1.99	1.94	1.93	1.73	1.64	1.61	1.03	0.07	0.07
cis-T_2	2.00	2.00	2.00	1.99	1.98	1.97	1.78	1.68	1.57	0.65	0.21	0.15
DHP-T_2	2.00	2.00	1.99	1.99	1.99	1.96	1.91	1.67	1.20	0.82	0.38	0.10

[a]H : HOMO, L : LUMO.

Table S2. The relative potential energies (in eV) calculated by SA6-CASSCF(18,12) for the *trans*-, *cis*-, *twist*-DANS and DHP.

	S_0	S_1	S_2	T_1	T_2	T_3
trans-S_0	0.00	5.18	5.53	3.07	4.59	4.97
cis-S_0	0.55	5.98	6.48	4.45	4.87	5.49
DHP-S_0	1.43	6.27	6.40	3.89	5.16	4.95
TS-S_0	2.53	4.92	5.69	2.51	5.67	5.81
trans-S_1	0.80	2.76	4.13	2.30	2.63	4.03
twist-S_1	2.85	4.50	5.86	2.85	5.82	6.01
DHP-S_1	2.93	4.52	5.35	3.29	5.03	5.77
trans-T_1	0.41	2.75	3.65	2.12	2.62	3.56
twist-T_1	2.50	5.05	5.68	2.47	5.66	5.73
DHP-T_1	2.56	4.78	5.73	2.74	5.44	6.35
trans-T_2	0.75	2.63	4.04	2.30	2.49	3.96
cis-T_2	0.90	5.98	6.24	4.16	4.44	5.71
DHP-T_2	2.33	5.18	5.85	3.75	4.52	5.88

S3. The occupation numbers of active orbitals and relative potential energies for CIs and ISCs.

Table S3. The occupation numbers of active orbitals calculated by SA6-CASSCF(18,12) for the conical intersections (CI) and intersystem crossings (ISC).[a]

	H-8[b]	H-7	H-6	H-5	H-4	H-3	H-2	H-1	H	L	L+1	L+2
CI-S_1/S_0-*trans*	2.00	2.00	1.99	1.99	1.99	1.94	1.91	1.65	1.33	0.82	0.28	0.09
CI-S_1/S_0-*cis*	2.00	2.00	2.00	2.00	1.99	1.95	1.92	1.63	1.33	0.81	0.29	0.08
CI-S_1/S_0-*twist-c*	2.00	2.00	2.00	2.00	1.99	1.97	1.94	1.76	1.21	0.70	0.37	0.06
CI-S_1/S_0-*twist-t*	2.00	2.00	2.00	2.00	1.99	1.96	1.95	1.71	1.28	0.72	0.35	0.05
CI-S_1/S_0-DHP	2.00	2.00	1.99	1.99	1.98	1.97	1.91	1.81	1.05	0.93	0.29	0.09
CI-T_2/T_1-*trans*	2.00	2.00	1.99	1.99	1.93	1.81	1.78	1.66	1.65	0.97	0.15	0.07
CI-T_2/T_1-*cis*	2.00	2.00	2.00	2.00	1.98	1.97	1.72	1.68	1.64	0.64	0.22	0.15
CI-T_2/T_1-*tict*	2.00	2.00	2.00	2.00	1.98	1.96	1.72	1.67	1.63	0.66	0.25	0.14
ISC-S_0/T_1-*twist*	2.00	2.00	2.00	1.99	1.99	1.99	1.84	1.74	1.18	0.92	0.21	0.15
ISC-S_1/T_1-*cis*	2.00	2.00	2.00	2.00	1.98	1.97	1.88	1.80	1.15	0.95	0.20	0.09
ISC-S_1/T_2-*trans*	2.00	2.00	1.99	1.99	1.94	1.93	1.75	1.61	1.59	1.06	0.08	0.07
ISC-S_1/T_2-*cis*	2.00	2.00	2.00	2.00	1.99	1.99	1.93	1.43	1.34	0.80	0.46	0.07
ISC-S_1/T_2-*twist*	2.00	2.00	1.99	1.99	1.98	1.95	1.86	1.40	1.14	0.98	0.66	0.06

[a]The energies are calculated by the BDF program on MOLPRO optimized geometries.
[b]H : HOMO, L : LUMO.

Table S4. The relative potential energies (in eV) calculated by SA6-CASSCF(18,12) for the conical intersections (CI) and intersystem crossings (ISC).[a]

	S_0	S_1	S_2	T_1	T_2	T_3
CI-S_1/S_0-*trans*	4.75	4.82	7.84	4.60	7.83	8.45
CI-S_1/S_0-*cis*	5.43	5.47	8.34	5.22	8.30	9.01
CI-S_1/S_0-*twist-c*	4.19	4.50	6.91	4.15	6.87	7.43
CI-S_1/S_0-*twist-t*	4.98	5.21	8.11	5.19	7.56	7.72
CI-S_1/S_0-DHP	4.07	4.07	5.16	3.90	6.43	6.73
CI-T_2/T_1-*trans*	1.12	2.66	4.38	2.51	2.51	4.29
CI-T_2/T_1-*cis*	0.97	6.26	6.44	4.43	4.44	5.79
CI-T_2/T_1-*tict*	0.63	5.95	6.36	3.81	4.36	5.47
ISC-S_0/T_1-*twist*	2.47	5.10	5.67	2.47	5.65	5.72
ISC-S_1/T_1-*cis*	3.94	4.27	6.60	4.03	6.94	7.19
ISC-S_1/T_2-*trans*	1.46	2.74	4.44	2.58	2.62	4.34
ISC-S_1/T_2-*cis*	1.47	4.55	5.27	2.60	4.55	5.24
ISC-S_1/T_2-*twist*	2.86	4.50	4.92	2.86	4.50	4.74

[a]The energies are calculated by the BDF program on MOLPRO optimized geometries.

S4. The LIICs for possible relaxation pathways calculated by SA6-CASSCF(18,12).

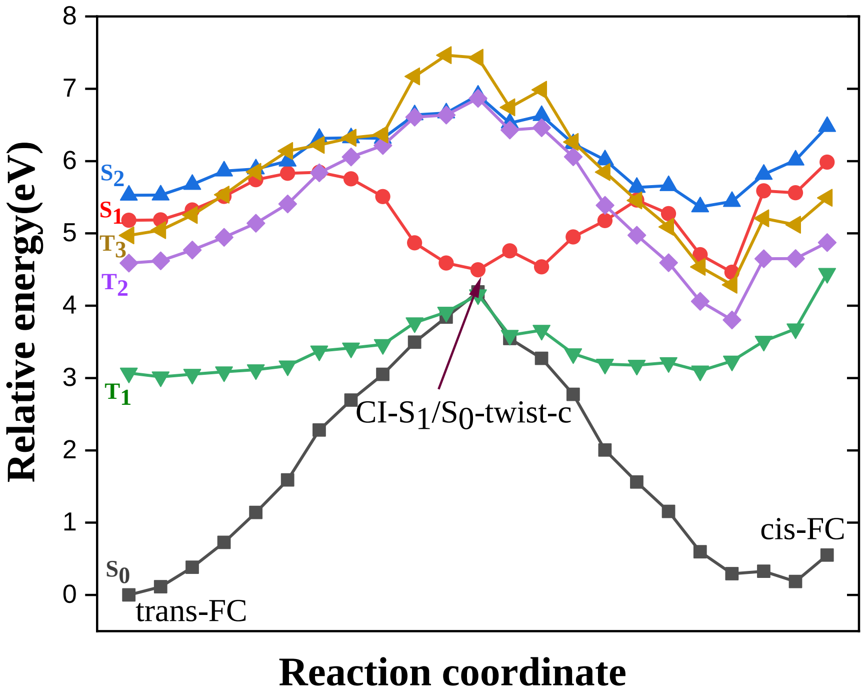

Figure S2a LIIC for *trans*-S_1-FC↔CI-S_1/S_0-*twist-c*↔cis-S_1-FC.

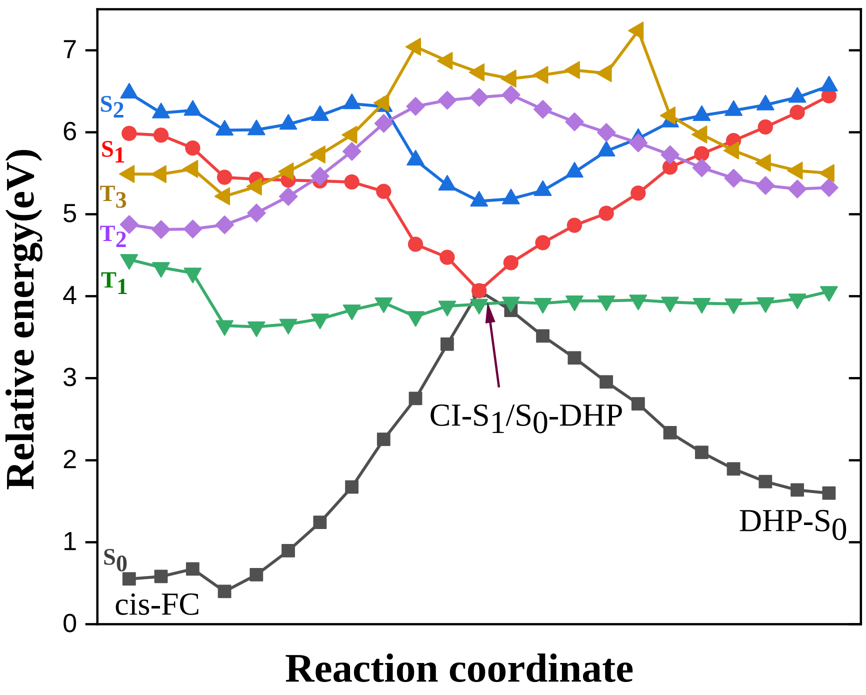

Figure S2b LIIC for *cis*-S_1-FC↔CI-S_1/S_0-DHP↔DHP-S_0.

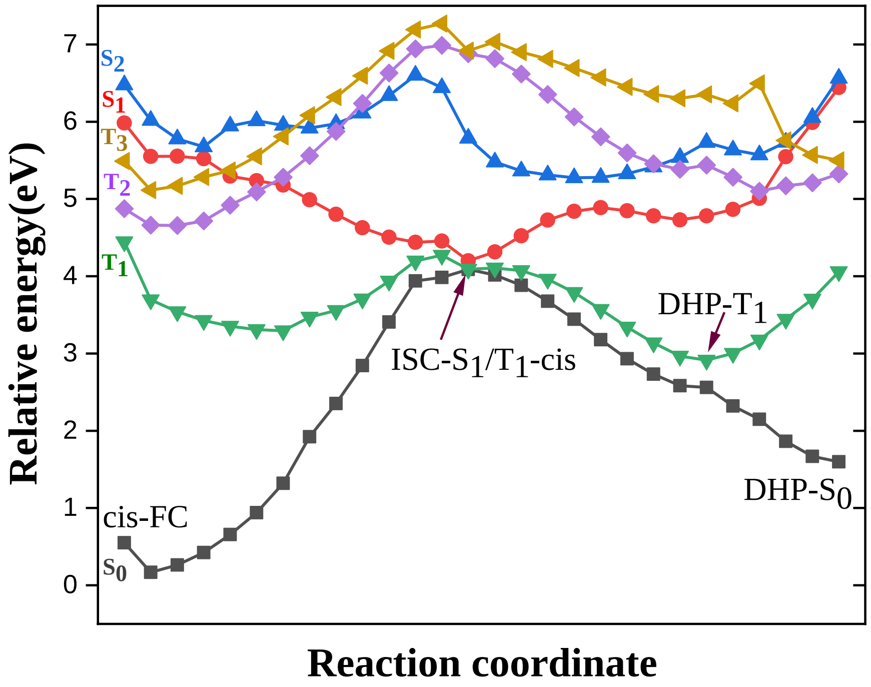

Figure S2c LIIC for *cis*-S_1-FC↔ISC-S_1/T_1-*cis*↔DHP-T_1↔DHP-S_0.

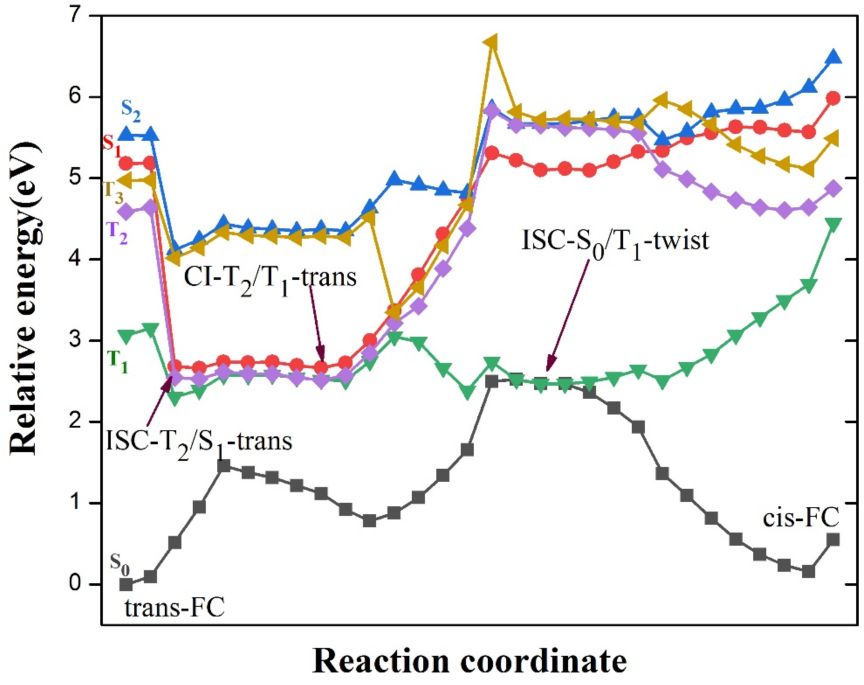

Figure S2d LIIC for *trans*-S_1-FC↔ISC-S_1/T_2-*trans*↔CI-T_2/T_1-*trans*↔ ISC-S_0/T_1-*twist*↔*cis*-S_1-FC.

S5. Geometries for SA6-CASSCF(18,12) optimized minima and TS of DANS.

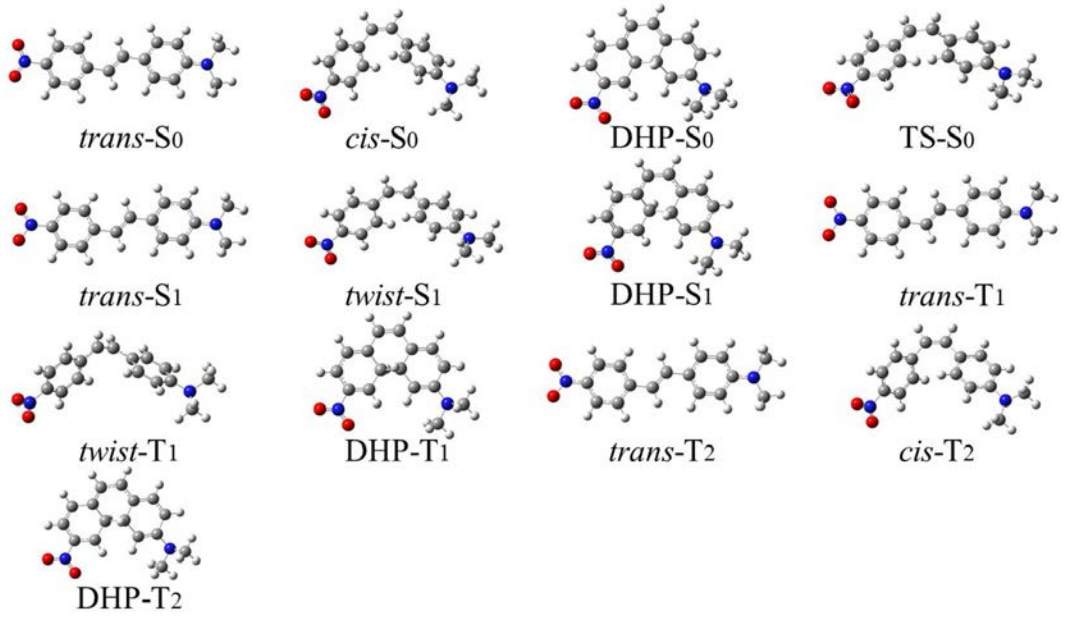

Figure S3. Geometries for SA6-CASSCF(18,12) optimized minima and TS of DANs.

S6. Cartesian coordinates for all optimized geometries (in Table 1 and Table 2).

***trans*-S_0**

C	0.002876	-0.000031	0.003522
C	0.005848	-0.000008	1.394419
C	1.184652	0.000005	2.122483
C	2.380888	0.000019	1.444063
C	2.436221	0.000014	0.039392
C	1.219472	-0.000010	-0.666920
C	3.755285	0.000033	-0.612135
C	3.990477	0.000046	-1.942946
C	5.301705	0.000065	-2.620886
C	5.342352	0.000121	-4.009405
C	6.530120	0.000029	-1.963683
C	6.528189	0.000135	-4.717964
C	7.725319	0.000044	-2.649394
C	7.760083	0.000093	-4.053902
H	-0.923951	0.000035	-0.532355
H	1.155813	-0.000002	3.193179
H	3.297487	0.000036	2.005520
H	1.211450	-0.000018	-1.739239
H	4.588590	0.000039	0.068061
H	3.151659	0.000043	-2.616487

H	4.418933	0.000154	-4.562933
H	6.570102	-0.000015	-0.889513
H	6.482948	0.000177	-5.788473
H	8.635575	0.000015	-2.083904
N	8.952058	0.000098	-4.743598
N	-1.271477	-0.000003	2.107119
O	-2.267990	0.000015	1.454950
O	-1.240649	-0.000019	3.297840
C	10.205436	0.000043	-4.027238
H	10.313180	-0.880142	-3.398617
H	11.020190	0.000048	-4.735914
H	10.313222	0.880180	-3.398557
C	8.950154	0.000145	-6.187237
H	8.456474	-0.879872	-6.591518
H	8.456502	0.880205	-6.591459
H	9.968973	0.000140	-6.545080

***cis*-S_0**

C	0.000000	0.000000	0.000000
C	0.000000	0.000000	1.387294
C	1.177549	0.000000	2.126444
C	2.386333	-0.004253	1.437649
C	2.419396	-0.024891	0.035122
C	1.203975	-0.010465	-0.666309
C	3.732225	0.010151	-0.658827
C	4.125454	-0.674367	-1.724294
C	3.401837	-1.729091	-2.476908
C	3.398090	-1.709308	-3.877659
C	2.746725	-2.785782	-1.847949
C	2.742496	-2.672588	-4.603703
C	2.106835	-3.770065	-2.574821
C	2.084474	-3.734521	-3.969452
H	-0.927203	0.012753	-0.535850
H	1.144600	0.006658	3.196483
H	3.307488	0.001796	1.991274
H	1.211935	-0.002566	-1.738782
H	4.443347	0.694627	-0.226653
H	5.111601	-0.450224	-2.098203
H	3.896983	-0.909240	-4.397146
H	2.752054	-2.850213	-0.775351
H	2.718182	-2.608544	-5.676190
H	1.628440	-4.571852	-2.045321
N	1.421775	-4.700302	-4.764969
N	-1.281833	0.009621	2.096797

O	-2.275501	0.013908	1.441513
O	-1.254390	0.011148	3.286913
C	0.397204	-5.488512	-4.117958
H	-0.276009	-4.843993	-3.567639
H	-0.178798	-6.002667	-4.878421
H	0.792043	-6.243340	-3.434304
C	2.290229	-5.511101	-5.599905
H	2.989507	-4.888743	-6.139854
H	2.860994	-6.241590	-5.022597
H	1.689342	-6.046018	-6.326233

DHP-S_0

C	-3.019441	2.320418	3.476328
C	-1.864928	2.944395	3.357590
C	-1.399365	3.553079	2.097069
C	-2.084115	3.331599	0.991576
C	-3.304306	2.502899	0.992166
C	-3.994472	2.242796	2.321608
H	-3.288737	1.895679	4.421473
H	-0.505239	4.141146	2.107292
H	-1.748189	3.737160	0.053775
H	-4.718498	3.049712	2.478910
C	-3.872506	2.051803	-0.160527
C	-5.080080	1.248507	-0.131880
H	-3.441019	2.310973	-1.111173
H	-5.594102	1.058419	-1.058048
C	-5.553035	0.742959	1.010350
C	-6.770836	-0.040324	1.053350
C	-4.774781	0.915415	2.310693
C	-7.369225	-0.360168	2.224507
H	-7.231436	-0.309913	0.119543
C	-5.646524	0.738303	3.538825
H	-4.030949	0.110629	2.320185
C	-6.819141	0.116847	3.513813
H	-8.309236	-0.876774	2.229360
H	-5.251003	1.087280	4.472417
N	-7.663808	-0.069005	4.629799
N	-0.977987	3.036881	4.514377
O	0.072708	3.611168	4.364251
O	-1.331137	2.543134	5.555757
C	-7.379609	0.729737	5.800136
H	-8.200238	0.625488	6.499886
H	-7.299673	1.772780	5.523611
H	-6.462877	0.434506	6.314716

C	-7.914833	-1.458610	4.973896
H	-7.045741	-1.933280	5.432847
H	-8.184048	-2.031864	4.097927
H	-8.741629	-1.510042	5.672236

TS-S$_0$

C	-1.455309	3.032514	3.187404
C	-0.326607	2.230584	3.062760
C	-0.273569	1.178743	2.148687
C	-1.359368	0.935589	1.356411
C	-2.534991	1.729727	1.444009
C	-2.543081	2.785676	2.391487
C	-3.647805	1.452927	0.602496
C	-4.896252	2.239857	0.563524
C	-6.055755	1.956204	1.327207
C	-7.234064	2.729438	1.175443
C	-6.126120	0.900508	2.276702
C	-8.383043	2.467679	1.903151
C	-7.259750	0.646486	2.993706
C	-8.432324	1.417260	2.842959
H	-1.463110	3.830739	3.901590
H	0.611390	0.579562	2.078497
H	-1.326782	0.128014	0.647706
H	-3.418575	3.398708	2.487659
H	-3.547862	0.631427	-0.088150
H	-4.945604	3.051522	-0.143556
H	-7.241457	3.539497	0.467331
H	-5.263970	0.279592	2.438555
H	-9.240654	3.086423	1.731507
H	-7.239194	-0.167285	3.691032
N	-9.572303	1.165711	3.609687
N	0.824387	2.493327	3.903603
O	0.755297	3.403020	4.676051
O	1.779543	1.785313	3.777507
C	-9.707385	-0.142667	4.216722
H	-10.650078	-0.189718	4.743265
H	-8.927837	-0.313922	4.947993
H	-9.681533	-0.954686	3.489646
C	-10.811524	1.797455	3.209507
H	-11.135885	1.510617	2.208473
H	-10.721082	2.875522	3.243349
H	-11.588145	1.522085	3.909023

***trans*-S$_1$**

C	-2.800660	-1.952696	0.037138
C	-1.412966	-1.890200	0.030475
C	-0.762877	-0.668803	0.023315
C	-1.516881	0.491180	0.022659
C	-2.908477	0.468700	0.028792
C	-3.529186	-0.782720	0.036423
C	-3.642448	1.752146	0.027352
C	-4.980131	1.914593	0.021487
C	-5.720851	3.187889	0.020186
C	-7.098808	3.173656	0.004630
C	-5.108273	4.455218	0.034300
C	-7.865165	4.339429	0.002090
C	-5.846853	5.619308	0.032063
C	-7.263776	5.590605	0.015545
H	-3.294736	-2.904932	0.043101
H	0.309280	-0.619210	0.018335
H	-1.004357	1.436473	0.017062
H	-4.599927	-0.855709	0.042427
H	-3.008722	2.621357	0.030427
H	-5.610334	1.042041	0.016274
H	-7.617398	2.230134	-0.006277
H	-4.037368	4.539430	0.047741
H	-8.932496	4.243367	-0.010568
H	-5.324349	6.554920	0.043478
N	-7.996852	6.756508	0.013325
N	-0.676899	-3.073707	0.031276
O	-1.114592	-4.252241	0.037465
O	0.643180	-3.114493	0.025858
C	-7.333582	8.039399	0.027484
H	-6.700372	8.179807	-0.844648
H	-8.076915	8.822719	0.022567
H	-6.721048	8.171191	0.915596
C	-9.438808	6.695521	-0.002914
H	-9.811446	6.189173	-0.889734
H	-9.830854	6.179715	0.869978
H	-9.839376	7.698417	-0.001937

***twist*-S$_1$**

C	-0.007445	-0.006048	0.002453
C	-0.005378	-0.000693	1.395451
C	1.230925	0.003695	2.077701
C	2.401683	-0.004113	1.398785
C	2.448796	-0.042251	-0.045198
C	1.158816	-0.029128	-0.704114

C	3.660461	-0.110418	-0.717945
C	3.783557	-0.065943	-2.155412
C	3.754629	-1.164014	-2.992584
C	3.894627	-1.035311	-4.415071
C	3.563047	-2.489595	-2.482832
C	3.864237	-2.101579	-5.239540
C	3.526837	-3.570496	-3.296184
C	3.673816	-3.427632	-4.711321
H	-0.947022	0.008554	-0.513942
H	1.229770	0.013809	3.149535
H	3.331201	-0.001934	1.939915
H	1.111383	-0.033059	-1.777838
H	4.577674	-0.011444	-0.159794
H	3.898326	0.887282	-2.662952
H	4.031314	-0.053417	-4.829885
H	3.441398	-2.601870	-1.425211
H	3.980075	-1.957093	-6.293423
H	3.378115	-4.539520	-2.868155
N	3.631530	-4.485127	-5.518038
N	-1.209804	0.002222	2.108069
O	-2.254628	-0.001741	1.494923
O	-1.171945	0.006150	3.317794
C	3.428419	-5.828618	-4.978217
H	2.479062	-5.898092	-4.462967
H	3.426745	-6.535703	-5.790833
H	4.225838	-6.094863	-4.296455
C	3.773706	-4.335892	-6.964735
H	2.985835	-3.713978	-7.369903
H	4.734736	-3.906663	-7.217654
H	3.706870	-5.307670	-7.424349

DHP-S_1

C	-3.244135	2.610583	3.462340
C	-1.847020	2.838413	3.419839
C	-1.170917	2.928537	2.242061
C	-1.839625	2.727852	1.009879
C	-3.183481	2.389158	0.956873
C	-4.015136	2.393446	2.219985
H	-3.763792	2.670761	4.394319
H	-0.117766	3.126142	2.248808
H	-1.268667	2.774528	0.100998
H	-4.745720	3.194217	2.131884
C	-3.829615	2.023993	-0.238526
C	-5.099994	1.485597	-0.239641

H	-3.313373	2.170822	-1.170585
H	-5.611492	1.343425	-1.175679
C	-5.759551	1.105928	0.965414
C	-7.113622	0.905424	1.033693
C	-4.912223	1.053580	2.205285
C	-7.773095	0.667038	2.238128
H	-7.696632	0.979583	0.131301
C	-5.694718	0.809635	3.449160
H	-4.171359	0.263351	2.092920
C	-7.031767	0.615904	3.466147
H	-8.842046	0.591186	2.261333
H	-5.131525	0.736437	4.359022
N	-7.773284	0.397282	4.646477
N	-1.131815	3.005531	4.663322
O	0.050400	3.248633	4.620737
O	-1.755537	2.892618	5.691357
C	-8.504941	-0.856740	4.701359
H	-9.227437	-0.812346	5.507624
H	-7.847679	-1.710658	4.876006
H	-9.042566	-1.031059	3.781097
C	-7.122707	0.698277	5.901310
H	-6.689778	1.689242	5.866962
H	-6.339729	-0.014484	6.167540
H	-7.866240	0.682908	6.689132

***trans*-T_1**

C	3.841395	-1.254805	-0.000069
C	4.695762	-0.154778	0.000007
C	4.169504	1.137256	0.000077
C	2.790736	1.304176	0.000074
C	1.910049	0.214488	0.000002
C	2.469614	-1.056775	-0.000071
C	0.456507	0.476750	0.000006
C	-0.516439	-0.428703	-0.000018
C	-1.968378	-0.185930	-0.000016
C	-2.840548	-1.269146	0.000030
C	-2.554390	1.079914	-0.000060
C	-4.213785	-1.118023	0.000038
C	-3.920470	1.254059	-0.000052
C	-4.796020	0.154968	-0.000002
H	4.242082	-2.249115	-0.000126
H	4.820158	1.989064	0.000134
H	2.393546	2.303548	0.000130
H	1.838688	-1.925512	-0.000134

H	0.199619	1.521952	0.000036
H	-0.252462	-1.472643	-0.000037
H	-2.439148	-2.268137	0.000062
H	-1.937673	1.959900	-0.000105
H	-4.824294	-1.998806	0.000075
H	-4.304849	2.254466	-0.000088
N	-6.163181	0.325379	0.000006
N	6.089683	-0.348458	0.000008
O	6.627590	-1.525154	-0.000048
O	6.929292	0.635847	0.000064
C	-6.729273	1.653376	-0.000041
H	-6.435416	2.219644	-0.880161
H	-7.806757	1.580903	-0.000029
H	-6.435400	2.219714	0.880028
C	-7.034124	-0.825684	0.000060
H	-6.885268	-1.446261	-0.879952
H	-6.885243	-1.446197	0.880112
H	-8.062164	-0.494948	0.000062

***twist*-T$_1$**

C	-1.274734	1.041046	2.624462
C	-0.472953	0.059665	2.043337
C	-0.821745	-0.547845	0.836072
C	-1.976633	-0.166445	0.213938
C	-2.822860	0.829010	0.767393
C	-2.429952	1.417776	1.995705
C	-4.022110	1.203275	0.093903
C	-4.946718	2.259274	0.541097
C	-6.063718	2.051995	1.398397
C	-6.924330	3.130295	1.737058
C	-6.379295	0.796620	1.967982
C	-7.993610	2.960307	2.563731
C	-7.466398	0.629561	2.789384
C	-8.307272	1.699871	3.110839
H	-0.980113	1.486441	3.552889
H	-0.185251	-1.299051	0.414663
H	-2.252321	-0.628428	-0.716807
H	-3.053672	2.168657	2.441872
H	-4.239239	0.700014	-0.834185
H	-4.790215	3.251131	0.150090
H	-6.713227	4.108625	1.341837
H	-5.757962	-0.052320	1.748627
H	-8.603613	3.805459	2.825368
H	-7.665864	-0.348132	3.184975

N	-9.421518	1.587642	3.970435
N	0.751918	-0.340844	2.709015
O	1.026348	0.196998	3.739465
O	1.416375	-1.186045	2.188363
C	-9.470787	0.432163	4.837291
H	-9.708093	-0.496810	4.314292
H	-10.236119	0.592927	5.587278
H	-8.524317	0.308846	5.347536
C	-10.709074	1.874405	3.362794
H	-11.451352	1.998964	4.142511
H	-11.041703	1.076022	2.696465
H	-10.667675	2.791832	2.793476

DHP-T_1

C	-3.183330	2.532195	3.477248
C	-1.878565	2.785798	3.428599
C	-1.142313	2.901541	2.203491
C	-1.842406	2.681847	1.002931
C	-3.164160	2.401640	0.973208
C	-4.007220	2.377876	2.232033
H	-3.675558	2.483756	4.426316
H	-0.095897	3.115193	2.216499
H	-1.290750	2.712486	0.079324
H	-4.694536	3.225367	2.198938
C	-3.858969	2.091456	-0.270863
C	-5.108117	1.537645	-0.268253
H	-3.366975	2.317861	-1.199521
H	-5.626168	1.376237	-1.196859
C	-5.771801	1.153118	0.966963
C	-7.098476	0.891028	1.016107
C	-4.915803	1.098363	2.212324
C	-7.764118	0.623075	2.220348
H	-7.675226	0.930020	0.107451
C	-5.714586	0.864492	3.466966
H	-4.224829	0.259762	2.102646
C	-7.031630	0.623767	3.472052
H	-8.828712	0.500453	2.235066
H	-5.161365	0.821216	4.384914
N	-7.799227	0.404466	4.638271
N	-1.144786	2.964055	4.681672
O	0.026399	3.239002	4.607376
O	-1.738511	2.828401	5.721253
C	-8.435321	-0.899749	4.714521
H	-9.181941	-0.887852	5.499756

H	-7.720580	-1.695864	4.931552
H	-8.932240	-1.140750	3.785615
C	-7.202340	0.787599	5.897454
H	-6.828786	1.801334	5.836011
H	-6.385394	0.133737	6.209283
H	-7.964346	0.754174	6.667183

***trans*-T_2**

C	3.830816	-1.260304	-0.000070
C	4.687798	-0.151092	0.000006
C	4.167961	1.144376	0.000078
C	2.787487	1.310457	0.000075
C	1.908284	0.219281	0.000002
C	2.473198	-1.064299	-0.000072
C	0.456353	0.477489	0.000006
C	-0.516140	-0.429082	-0.000018
C	-1.967927	-0.186234	-0.000016
C	-2.840240	-1.269426	0.000030
C	-2.553908	1.079840	-0.000060
C	-4.213337	-1.118253	0.000038
C	-3.919861	1.254044	-0.000052
C	-4.795694	0.154918	-0.000002
H	4.238495	-2.252927	-0.000126
H	4.823703	1.993444	0.000134
H	2.388361	2.308734	0.000132
H	1.839723	-1.930825	-0.000134
H	0.198083	1.522272	0.000036
H	-0.252318	-1.473085	-0.000038
H	-2.439013	-2.268500	0.000061
H	-1.937466	1.960030	-0.000104
H	-4.823834	-1.998992	0.000075
H	-4.304243	2.254440	-0.000088
N	-6.162414	0.325293	0.000006
N	6.053500	-0.345663	0.000008
O	6.663284	-1.467391	-0.000051
O	6.949404	0.567540	0.000067
C	-6.728878	1.653366	-0.000042
H	-6.435228	2.219578	-0.880207
H	-7.806307	1.580568	-0.000029
H	-6.435213	2.219647	0.880074
C	-7.033736	-0.825761	0.000060
H	-6.885064	-1.446242	-0.879995
H	-6.885039	-1.446177	0.880155
H	-8.061645	-0.494733	0.000062

cis-T_2

C	-1.778075	2.906814	3.247124
C	-0.778616	1.891410	3.229929
C	-0.713688	0.934332	2.170578
C	-1.646686	1.006705	1.156720
C	-2.665812	2.003578	1.158359
C	-2.677872	2.953289	2.245682
C	-3.613563	2.117236	0.024306
C	-4.949626	2.143383	0.048829
C	-5.887983	1.976320	1.145539
C	-7.222541	2.495218	1.006523
C	-5.592607	1.293964	2.378372
C	-8.137528	2.384770	1.989566
C	-6.513017	1.175730	3.360996
C	-7.831440	1.716846	3.225939
H	-1.793700	3.613821	4.050585
H	0.051948	0.187251	2.181117
H	-1.611066	0.295644	0.352237
H	-3.435051	3.714629	2.244850
H	-3.144122	2.221996	-0.940362
H	-5.422190	2.325185	-0.903371
H	-7.481557	3.012857	0.099435
H	-4.631913	0.835972	2.509417
H	-9.107584	2.828860	1.865843
H	-6.251119	0.638117	4.252346
N	-8.794924	1.657921	4.235787
N	0.182834	1.829167	4.289679
O	0.100293	2.641954	5.164913
O	1.012896	0.967299	4.239954
C	-8.359139	1.322073	5.572275
H	-8.125625	0.263166	5.697378
H	-9.150196	1.571021	6.269417
H	-7.486489	1.903420	5.838182
C	-10.078282	1.067861	3.898661
H	-10.814445	1.365012	4.636146
H	-10.035464	-0.022190	3.875074
H	-10.417488	1.410464	2.932466

DHP-T_2

C	-3.065548	2.257897	3.484041
C	-1.737110	2.755144	3.385243
C	-1.142383	3.073817	2.172242
C	-1.831694	2.887276	1.003894

C	-3.195356	2.377710	0.974949
C	-3.973832	2.280168	2.282867
C	-3.472325	2.082768	4.454944
C	-0.136981	3.442384	2.159015
C	-1.350929	3.096918	0.066204
C	-4.581648	3.187922	2.361463
C	-3.783711	2.035547	-0.176623
C	-5.142615	1.539205	-0.214803
C	-3.253972	2.154367	-1.106217
C	-5.669557	1.510617	-1.150425
H	-5.782187	1.148393	0.961309
H	-7.119962	0.811947	0.993527
H	-4.944783	1.078097	2.229790
H	-7.787005	0.531397	2.185547
H	-7.679735	0.822857	0.074549
H	-5.780078	0.892592	3.474433
H	-4.311076	0.190590	2.134738
H	-7.085452	0.607491	3.457161
H	-8.844471	0.355576	2.182576
H	-5.257861	0.918554	4.409857
N	-7.881299	0.421885	4.610800
N	-0.971696	2.908583	4.594434
O	0.159904	3.326100	4.510346
O	-1.497531	2.610959	5.642027
C	-8.470631	-0.900493	4.735147
H	-9.234323	-0.880205	5.503528
H	-7.732355	-1.658568	5.003335
H	-8.938519	-1.202622	3.808784
C	-7.330786	0.885317	5.864227
H	-6.988664	1.906759	5.762203
H	-6.501255	0.275071	6.227186
H	-8.111161	0.862725	6.615579

CI-S_1/S_0-*trans*

C	0.361257	-0.496633	0.465304
C	0.182291	-0.204652	1.890085
C	-0.560153	0.882265	2.315653
C	-0.774296	1.118447	3.679587
C	-0.251633	0.289831	4.639136
C	0.503193	-0.821546	4.217061
C	0.708243	-1.068828	2.875180
C	0.694087	0.396320	-0.559789
H	1.529463	-0.519624	0.310119
H	1.361139	1.243312	-0.389176

C	0.354839	0.199268	-1.971595
C	0.896566	1.052587	-2.945044
C	0.555241	0.919502	-4.268392
C	-0.334291	-0.089965	-4.623333
C	-0.883689	-0.959330	-3.685112
C	-0.533303	-0.815733	-2.365990
H	-0.990580	1.552884	1.593692
H	-1.355466	1.967301	3.991174
H	0.918913	-1.494621	4.945772
H	1.265102	-1.936806	2.569646
H	1.583333	1.826442	-2.652625
H	0.957664	1.568687	-5.018634
H	-1.564361	-1.723735	-3.999850
H	-0.923052	-1.475262	-1.615963
N	-0.698971	-0.244614	-6.030563
N	-0.497446	0.573434	6.019468
O	-1.455259	-1.121018	-6.310510
O	-0.217410	0.516511	-6.810788
C	0.696943	0.878365	6.781918
H	0.411152	1.251307	7.759679
H	1.352761	0.017152	6.930975
H	1.262836	1.651548	6.277592
C	-1.347504	-0.400616	6.676204
H	-1.626142	-0.028998	7.656564
H	-2.251851	-0.542223	6.098229
H	-0.871273	-1.375041	6.809177

CI-S_1/S_0-*cis*

C	-0.119041	0.334594	0.132360
C	-0.030994	0.253549	-1.321206
C	0.854214	1.039841	-2.032082
C	0.881167	1.027325	-3.435336
C	0.042680	0.216571	-4.161803
C	-0.854777	-0.601063	-3.442444
C	-0.905827	-0.571558	-2.066660
C	0.810574	0.206765	1.173319
H	-0.107802	-0.767178	0.567558
H	0.556787	0.676282	2.115489
C	2.038139	-0.627341	1.279338
C	2.751558	-0.602854	2.485211
C	3.930414	-1.294750	2.628046
C	4.401553	-2.029889	1.546670
C	3.719173	-2.079342	0.335519
C	2.547374	-1.377012	0.203931

H	1.530719	1.690935	-1.507683
H	1.583087	1.671180	-3.931274
H	-1.502813	-1.265946	-3.983901
H	-1.615881	-1.193003	-1.550014
H	2.372787	-0.033231	3.314482
H	4.478101	-1.280737	3.548017
H	4.113693	-2.654763	-0.476908
H	2.023098	-1.398034	-0.731181
N	5.651321	-2.773762	1.687445
N	0.041084	0.133808	-5.577608
O	6.215696	-2.708184	2.734639
O	6.028555	-3.400003	0.747203
C	-1.164013	0.640106	-6.210768
H	-1.173455	0.341951	-7.253061
H	-1.233384	1.729089	-6.163788
H	-2.043878	0.227347	-5.737561
C	1.236257	0.583560	-6.253410
H	1.192052	0.267803	-7.289431
H	2.109745	0.131652	-5.801081
H	1.363067	1.668659	-6.243329

CI-S_1/S_0-*twist-c*

C	0.000000	0.000000	0.000000
C	0.000000	0.000000	1.397944
C	1.266420	0.000000	2.047927
C	2.423613	0.010389	1.366464
C	2.481273	0.003903	-0.106406
C	1.147254	0.005613	-0.729107
C	3.646952	-0.032950	-0.786008
C	3.554208	0.437572	-2.144705
C	3.637595	-0.326749	-3.264529
C	3.503576	0.232287	-4.580454
C	3.791515	-1.756208	-3.142725
C	3.512776	-0.542049	-5.685465
C	3.813102	-2.550433	-4.234741
C	3.659380	-1.980841	-5.557601
H	-0.944685	-0.005537	-0.511528
H	1.289764	-0.000712	3.121723
H	3.359995	0.003489	1.897062
H	1.054498	-0.039851	-1.801675
H	4.526404	0.380063	-0.310345
H	3.380236	1.493913	-2.401312
H	3.395743	1.295576	-4.681069
H	3.866001	-2.126342	-2.141922

H	3.430409	-0.098663	-6.653884
H	3.895274	-3.609503	-4.121176
N	3.646510	-2.743065	-6.613592
N	-1.201057	-0.017337	2.128512
O	-2.261561	-0.023242	1.533227
O	-1.157189	-0.025525	3.341783
C	3.910168	-4.191030	-6.534916
H	3.036548	-4.716396	-6.174788
H	4.152497	-4.542142	-7.523706
H	4.753819	-4.390168	-5.895025
C	3.354159	-2.218110	-7.958020
H	2.525441	-1.530086	-7.927478
H	4.226633	-1.734209	-8.375870
H	3.073863	-3.045022	-8.588252

CI-S_1/S_0-*twist-t*

C	-5.474772	-1.269659	-1.971333
C	-4.694419	-1.664698	-3.028078
C	-3.267473	-1.630912	-2.867095
C	-2.665419	-1.025941	-1.826117
C	-3.475482	-0.307897	-0.852446
C	-4.916779	-0.645399	-0.855013
H	-6.525217	-1.491486	-1.985966
H	-2.673056	-2.119418	-3.615185
H	-1.594014	-1.024782	-1.730447
H	-5.446382	-0.698851	0.077733
C	-3.119024	0.746523	-0.069452
C	-4.363065	1.490770	-0.185240
H	-2.179735	1.247970	-0.192422
H	-5.142202	1.381740	0.552022
C	-4.620575	2.550921	-1.081421
C	-5.850154	3.254337	-1.011157
C	-3.708189	2.942844	-2.096575
C	-6.147461	4.278164	-1.848522
H	-6.569786	2.965256	-0.267061
C	-3.983800	3.957736	-2.948576
H	-2.788994	2.402600	-2.200969
C	-5.223971	4.668741	-2.866524
H	-7.087766	4.778978	-1.750763
H	-3.270429	4.209503	-3.704953
N	-5.509147	5.657203	-3.717737
N	-5.264446	-2.250141	-4.158428
O	-4.535555	-2.691111	-5.018815
O	-6.471702	-2.288298	-4.257172

C	-4.567005	6.037317	-4.766532
H	-4.977329	6.864325	-5.321899
H	-3.621989	6.351558	-4.342477
H	-4.395864	5.217329	-5.452715
C	-6.792462	6.349447	-3.654182
H	-7.613496	5.660999	-3.809996
H	-6.918756	6.845832	-2.700157
H	-6.826448	7.096450	-4.429682

CI-S_1/S_0-DHP

C	-3.301819	2.937999	3.246282
C	-1.931892	3.040948	3.282673
C	-1.169086	2.983976	2.075745
C	-1.778475	2.708708	0.897897
C	-3.169187	2.406415	0.820659
C	-3.977277	2.589171	2.039631
H	-3.875560	3.104820	4.136333
H	-0.112000	3.151977	2.118792
H	-1.189992	2.656285	-0.000800
H	-4.925905	3.078394	1.901043
C	-3.751696	1.890337	-0.340037
C	-5.035740	1.330022	-0.320568
H	-3.211870	1.950135	-1.268071
H	-5.569606	1.225909	-1.251806
C	-5.648515	0.899544	0.843333
C	-7.058156	0.707163	0.932624
C	-4.859452	0.812321	2.080349
C	-7.693845	0.497015	2.100662
H	-7.634012	0.776967	0.025354
C	-5.569689	0.535126	3.298004
H	-3.911819	0.307416	1.979106
C	-6.935736	0.414905	3.338600
H	-8.758752	0.394849	2.114315
H	-4.982269	0.376586	4.179378
N	-7.614796	0.187681	4.508321
N	-1.255992	3.297230	4.530868
O	-0.065409	3.497615	4.499345
O	-1.908018	3.298559	5.546784
C	-8.949515	-0.384008	4.482871
H	-9.248221	-0.615000	5.494480
H	-8.994657	-1.301665	3.903199
H	-9.675535	0.313515	4.082032
C	-6.879097	0.099540	5.751225
H	-6.230597	0.958157	5.865578

H	-6.271410	-0.801625	5.817612
H	-7.576227	0.102948	6.576704

CI-T$_2$/T$_1$-*trans*

C	-2.045497	-1.581904	1.247296
C	-1.478298	-1.893250	0.009343
C	-1.711374	-1.078374	-1.111247
C	-2.519059	0.035284	-0.972019
C	-3.117208	0.360774	0.260898
C	-2.850680	-0.458936	1.348574
C	-3.997498	1.530994	0.439744
C	-4.633215	2.187878	-0.524700
C	-5.509953	3.361118	-0.357646
C	-6.387969	3.708118	-1.373309
C	-5.500717	4.173230	0.781278
C	-7.251330	4.788984	-1.260051
C	-6.342100	5.254514	0.895593
C	-7.249678	5.581420	-0.119462
H	-1.858552	-2.204271	2.101159
H	-1.256640	-1.314158	-2.054211
H	-2.670214	0.669146	-1.824752
H	-3.287090	-0.225175	2.303228
H	-4.138630	1.834684	1.462915
H	-4.542736	1.839078	-1.539674
H	-6.418384	3.116267	-2.271816
H	-4.802271	3.978521	1.574367
H	-7.922718	4.997778	-2.070232
H	-6.280157	5.881495	1.765641
N	-8.078621	6.710921	0.041038
N	-0.677612	-2.997838	-0.113261
O	-0.343155	-3.841635	0.767872
O	-0.082195	-3.430209	-1.157088
C	-8.716362	7.236371	-1.144477
H	-7.987439	7.365105	-1.933897
H	-9.132399	8.209425	-0.912380
H	-9.527520	6.607733	-1.518145
C	-8.980618	6.675365	1.179167
H	-8.460443	6.364103	2.072832
H	-9.820367	5.995873	1.020934
H	-9.374695	7.669817	1.352221

CI-T$_2$/T$_1$-*cis*

C	-1.232634	2.972220	-3.066236
C	-0.758479	1.628108	-3.186914

C	-1.227240	0.597723	-2.319071
C	-2.135109	0.907294	-1.374176
C	-2.655328	2.247747	-1.214104
C	-2.160710	3.262786	-2.089235
C	-3.603886	2.564414	-0.119813
C	-4.943609	2.617632	-0.153178
C	-5.894090	2.379170	-1.211065
C	-7.292581	2.550803	-0.923821
C	-5.572175	1.976506	-2.555714
C	-8.255898	2.335741	-1.848501
C	-6.534445	1.762330	-3.476914
C	-7.927127	1.916365	-3.173290
H	-0.858496	3.723229	-3.729978
H	-0.850098	-0.397265	-2.434787
H	-2.499125	0.141277	-0.714335
H	-2.528775	4.265846	-1.984042
H	-3.137994	2.783270	0.828440
H	-5.405454	2.886302	0.783021
H	-7.578278	2.853700	0.068102
H	-4.548475	1.856341	-2.845905
H	-9.283868	2.467192	-1.569662
H	-6.252439	1.497815	-4.479092
N	-8.878496	1.704159	-4.173809
N	0.204895	1.313561	-4.197652
O	0.577665	2.201688	-4.909589
O	0.582001	0.179375	-4.273488
C	-8.870335	0.410362	-4.833980
H	-9.337808	-0.364102	-4.223872
H	-9.411995	0.481842	-5.769640
H	-7.861019	0.097888	-5.056134
C	-10.213503	2.214613	-3.960461
H	-10.747199	2.197367	-4.903026
H	-10.789751	1.631005	-3.240103
H	-10.171585	3.241062	-3.621299

CI-T_2/T_1-tict

C	-3.477770	-2.392582	0.697095
C	-2.343901	-2.524918	-0.191904
C	-1.836106	-1.381634	-0.919176
C	-2.434033	-0.195106	-0.758291
C	-3.579319	-0.019474	0.120289
C	-4.054083	-1.192574	0.836177
C	-4.204014	1.306421	0.314384
C	-5.277048	1.746186	-0.438391

C	-5.960729	3.025244	-0.321417
C	-7.022398	3.312944	-1.180477
C	-5.628386	4.023360	0.614333
C	-7.714037	4.495843	-1.133408
C	-6.323335	5.228390	0.671322
C	-7.376837	5.501901	-0.192999
H	-3.827952	-3.257602	1.220373
H	-0.996178	-1.513755	-1.568609
H	-2.071876	0.664254	-1.292093
H	-4.897640	-1.075738	1.491377
H	-3.782826	1.933006	1.082588
H	-5.656576	1.081347	-1.195726
H	-7.315082	2.580095	-1.913045
H	-4.824150	3.871254	1.309366
H	-8.517946	4.646812	-1.825453
H	-6.022496	5.947993	1.406651
N	-8.069656	6.692819	-0.149439
N	-1.725024	-3.785256	-0.351632
O	-2.174026	-4.713359	0.267740
O	-0.784689	-3.858046	-1.098006
C	-7.719310	7.690693	0.832478
H	-6.692061	8.025847	0.716110
H	-7.841246	7.322683	1.848570
H	-8.361318	8.550718	0.712858
C	-9.180995	6.925102	-1.041861
H	-9.978467	6.199825	-0.899958
H	-8.876600	6.894192	-2.084546
H	-9.590719	7.905607	-0.850019

ISC-S_0/T_1-*twist*

C	-1.173940	-0.682423	-2.580281
C	-1.058415	0.188725	-3.662623
C	-0.002967	1.096708	-3.760165
C	0.935979	1.126811	-2.769061
C	0.860617	0.258162	-1.648856
C	-0.230304	-0.646668	-1.591164
H	-1.997137	-1.366054	-2.534700
H	0.058022	1.753215	-4.604094
H	1.753028	1.821985	-2.836277
H	-0.317337	-1.311108	-0.753096
C	1.855966	0.316347	-0.631532
C	1.916960	-0.569731	0.544573
H	2.650551	1.032173	-0.765799
H	2.551299	-1.437573	0.474784

C	1.238729	-0.339239	1.774806
C	1.382909	-1.246857	2.858982
C	0.386405	0.767831	1.993505
C	0.735827	-1.056630	4.042142
H	2.007073	-2.114269	2.732225
C	-0.252193	0.960576	3.194085
H	0.237898	1.482897	1.205214
C	-0.097691	0.061149	4.253240
H	0.841306	-1.780677	4.829246
H	-0.878071	1.825051	3.309240
N	-0.750693	0.198339	5.497147
N	-2.060040	0.151971	-4.712833
O	-1.935431	0.911809	-5.625370
O	-2.950617	-0.635486	-4.603939
C	0.122747	0.382491	6.643040
H	-0.445136	0.228685	7.553179
H	0.559898	1.382483	6.675965
H	0.929271	-0.336382	6.628267
C	-1.904222	1.068109	5.543080
H	-2.432394	0.896484	6.473530
H	-2.578200	0.835593	4.728837
H	-1.652973	2.129866	5.494811

ISC-S_1/T_1-*cis*

C	-3.276011	3.103324	3.186178
C	-1.915595	3.139476	3.264803
C	-1.118994	3.015799	2.054161
C	-1.708036	2.727085	0.876085
C	-3.138789	2.422076	0.797714
C	-3.928607	2.758964	1.998183
H	-3.859741	3.345527	4.053020
H	-0.061199	3.171863	2.119535
H	-1.120407	2.640722	-0.020076
H	-4.939028	3.093901	1.871006
C	-3.668641	1.797001	-0.274380
C	-5.068231	1.359368	-0.276537
H	-3.069750	1.614928	-1.147526
H	-5.655991	1.466731	-1.173832
C	-5.665230	0.862425	0.849188
C	-7.095878	0.712965	0.948770
C	-4.878141	0.674213	2.089022
C	-7.716203	0.456330	2.104694
H	-7.677288	0.860313	0.055578
C	-5.556911	0.354261	3.282375

H	-3.878001	0.308607	1.973671
C	-6.939735	0.278298	3.337245
H	-8.783912	0.398482	2.138310
H	-4.969144	0.142952	4.151527
N	-7.593786	0.013259	4.481543
N	-1.281245	3.404319	4.500414
O	-0.084248	3.527110	4.509885
O	-1.959093	3.491282	5.494604
C	-9.009441	-0.329800	4.499110
H	-9.251126	-0.747759	5.464067
H	-9.244273	-1.076498	3.752317
H	-9.638100	0.540071	4.340167
C	-6.875789	-0.022377	5.746545
H	-6.215480	0.829417	5.831514
H	-6.291670	-0.931337	5.853699
H	-7.588112	0.026637	6.555935

ISC-S_1/T_2-*trans*

C	-2.832052	-1.943969	0.023271
C	-1.424172	-1.889569	0.030739
C	-0.766502	-0.657103	0.036911
C	-1.521124	0.500006	0.035505
C	-2.912118	0.480927	0.028160
C	-3.544424	-0.779710	0.022107
C	-3.641686	1.759084	0.027118
C	-4.961605	1.926453	0.020106
C	-5.703151	3.196856	0.018980
C	-7.093968	3.174194	0.010698
C	-5.104979	4.457435	0.025755
C	-7.856182	4.326005	0.009121
C	-5.845001	5.618499	0.024338
C	-7.250227	5.588319	0.015942
H	-3.330409	-2.894520	0.018597
H	0.305578	-0.614682	0.042625
H	-1.009214	1.445617	0.040269
H	-4.615724	-0.842242	0.016411
H	-3.004479	2.626336	0.032726
H	-5.595100	1.055854	0.014429
H	-7.605027	2.226622	0.005252
H	-4.034161	4.546957	0.032350
H	-8.923620	4.231755	0.002551
H	-5.322208	6.553989	0.029833
N	-7.993541	6.747341	0.014531
N	-0.704248	-3.046266	0.031983

O	-1.083880	-4.236273	0.027294
O	0.648344	-3.166400	0.038650
C	-7.335401	8.032582	0.021482
H	-6.707363	8.171340	-0.854741
H	-8.080793	8.813909	0.019135
H	-6.717516	8.166970	0.905562
C	-9.435758	6.680610	0.005557
H	-9.812016	6.171767	-0.878204
H	-9.822712	6.167096	0.881961
H	-9.838898	7.682404	0.005776

ISC-S_1/T_2-*cis*

C	-1.977198	2.693075	2.693075
C	-0.723111	2.100839	2.100839
C	-0.406000	1.402602	1.402602
C	-1.323578	1.281087	1.281087
C	-2.656567	1.827078	1.827078
C	-2.913985	2.574760	2.574760
C	-3.571722	1.635220	1.635220
C	-4.950943	2.054936	2.054936
C	-5.904177	1.845851	1.845851
C	-7.204086	2.443231	2.443231
C	-5.654777	1.043796	1.043796
C	-8.130088	2.301910	2.301910
C	-6.590378	0.890834	0.890834
C	-7.853071	1.523523	1.523523
H	-2.199926	3.259841	3.259841
H	0.568023	0.967876	0.967876
H	-1.068414	0.741570	0.741570
H	-3.848678	3.086303	3.086303
H	-3.216411	1.190312	1.190312
H	-5.308889	2.550532	2.550532
H	-7.441388	3.036553	3.036553
H	-4.718889	0.531052	0.531052
H	-9.081164	2.780966	2.780966
H	-6.353552	0.269703	0.269703
N	-8.773208	1.397895	1.397895
N	0.230762	2.217015	2.217015
O	-0.053619	2.823911	2.823911
O	1.351675	1.708219	1.708219
C	-8.474325	0.617238	0.617238
H	-8.305203	-0.426715	-0.426715
H	-9.310079	0.672580	0.672580
H	-7.598936	1.000835	1.000835

C	-10.075494	2.030183	2.030183
H	-9.987714	3.108560	3.108560
H	-10.655235	1.804780	1.804780
H	-10.621093	1.663145	1.663145

ISC-S_1/T_2-*twist*

C	-3.051559	-0.866257	1.029448
C	-1.686547	-1.080148	0.859888
C	-1.002880	-0.442164	-0.171954
C	-1.686043	0.396048	-1.018832
C	-3.064528	0.634186	-0.879506
C	-3.721460	-0.024722	0.173707
C	-3.743754	1.512509	-1.778127
C	-5.189201	1.780764	-1.762043
C	-5.799170	2.939235	-1.147181
C	-7.185775	3.163278	-1.266644
C	-5.083880	3.874735	-0.415713
C	-7.803424	4.254158	-0.690104
C	-5.693834	4.980638	0.171057
C	-7.061502	5.203279	0.052720
H	-3.576150	-1.355750	1.826122
H	0.048494	-0.606331	-0.302576
H	-1.148277	0.882570	-1.813147
H	-4.773249	0.135904	0.319180
H	-3.149757	1.982728	-2.545945
H	-5.834303	1.112887	-2.309296
H	-7.785355	2.465183	-1.824900
H	-4.023812	3.750498	-0.287535
H	-8.861288	4.370197	-0.817279
H	-5.076692	5.661705	0.722728
N	-7.677306	6.294705	0.628725
N	-1.000889	-1.935035	1.726413
O	-1.582639	-2.555147	2.700442
O	0.265429	-2.170106	1.617562
C	-6.895361	7.245063	1.381869
H	-6.128208	7.712816	0.769796
H	-7.542449	8.025699	1.753847
H	-6.408353	6.781656	2.236301
C	-9.100224	6.490766	0.485004
H	-9.393146	6.599993	-0.556132
H	-9.669925	5.669140	0.911776
H	-9.388323	7.393515	1.003111
